# Supplementary material for: Vagus nerve inflammation contributes to dysautonomia in COVID-19
Source: Acta Neuropathol. 2023 Jul 15;146(3):387–94. doi: 10.1007/s00401-023-02612-x (PMC10412500; doi:10.1007/s00401-023-02612-x)
Supplement: Supplementary file 1 — (DOCX 400 KB) [file 401_2023_2612_MOESM1_ESM.docx]

**Supplementary figures**

**
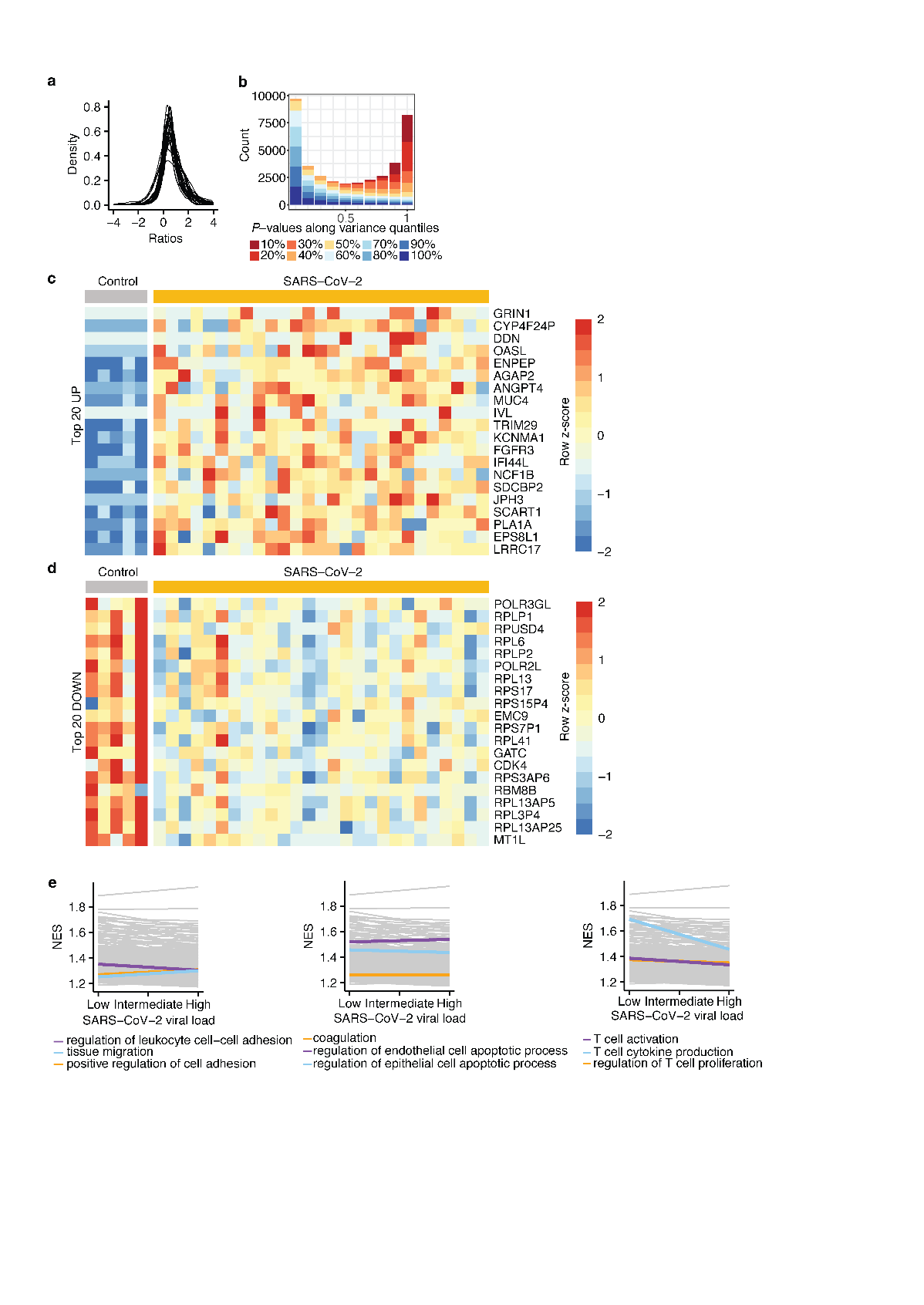
**

**Sup Fig 1. RNA sequencing of vagus nerves. (a-b)** Quality control of RNA sequencing experiments. Density distribution of detected genes (a) and *P* values of genes along the variance quantiles (b). **(c-d)** Top 20 upregulated (c) and downregulated (d) genes in vagus nerves of COVID-19 patients in comparison to controls. Color shows row z-score. **(e)** GO terms that are significantly upregulated independently of SARS-CoV-2 viral load.


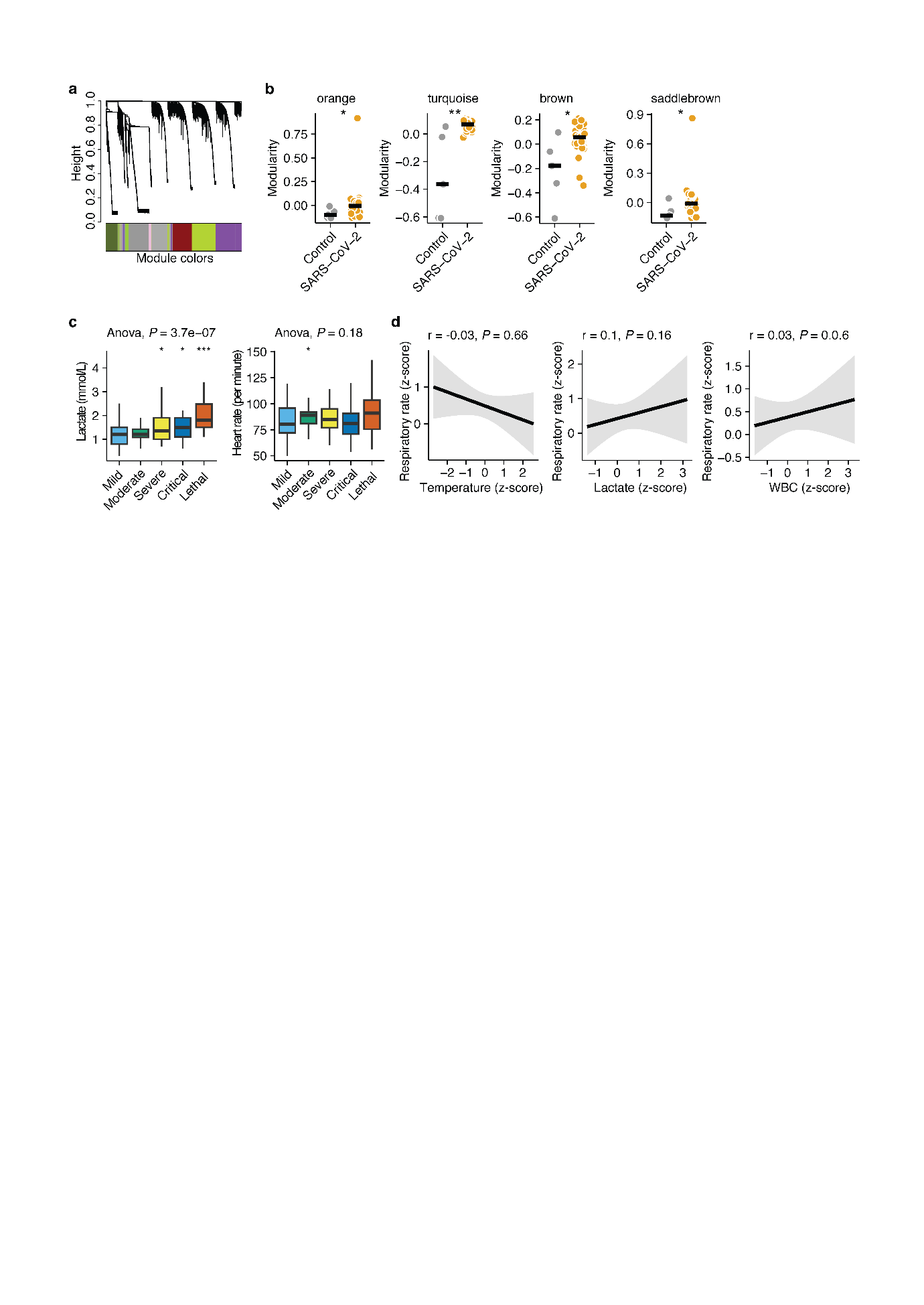


**Sup Fig 2. Respiratory rate is decoupled from inflammation in COVID-19. (a)** Dendrogram of weighted gene correlated network analysis (WGCNA) for module detection. **(b)** Comparison of module enrichment by COVID-19 samples in orange, turquoise, brown and saddlebrown modules. Wilcoxon-test was used for statistical comparison. **P* < 0.05, ***P* < 0.001. **(c)** Comparison of lactate and heart rate in patients with mild, moderate, critical, and lethal COVID-19. One-way ANOVA was performed for testing group differences. *T*-test with FDR-correction for multiple comparisons against mild COVID-19 group was performed. **P* < 0.05, ***P* < 0.01, ****P* < 0.001. **(d)** Correlation analyses of z-transformed respiratory rate and z-transformed temperature, lactate, and white blood cell count (WBC). Spearman correlation analysis was performed. Correlation coefficients and *P*-values are provided in the figure.

**Supplementary tables**

**Sup Table 1. Patients’ demographics for vagus nerve analyses.**

| Cases | Age range | Sex | PMI (days) | Cause of death | RNA | IHC |
| --- | --- | --- | --- | --- | --- | --- |
| COVID | 71-80 | m | 2 | Pneumonia | X | X |
| COVID | 91-100 | m | 5 | Sepsis, Pneumonia | X | X |
| COVID | 81-90 | w | 3 | Pneumonia | X | X |
| COVID | 91-100 | m | 3 | Acute Myocardial Infarction | X | X |
| COVID | 71-80 | m | 3 | Pneumonia |  | X |
| COVID | 81-90 | m | 3 | Pneumonia | X | X |
| COVID | 91-100 | w | 3 | Pulmonary embolism | X | X |
| COVID | 91-100 | w | 2 | Pneumonia |  | X |
| COVID | 71-80 | m | 1 | Pneumonia | X | X |
| COVID | 81-90 | m | 4 | Pneumonia | X | X |
| COVID | 81-90 | m | 2 | Acute exacerbated COPD | X | X |
| COVID | 81-90 | w | 3 | Pneumonia | X | X |
| COVID | 81-90 | m | 4 | Pneumonia |  | X |
| COVID | 81-90 | w | 3 | Pneumonia | X | X |
| COVID | 81-90 | m | 2 | Sepsis in urocystitis and pneumonia | X | X |
| COVID | 41-50 | m | 1 | Brain mass hemorrhage under ECMO in pneumonia | X | X |
| COVID | 81-90 | m | 3 | Myocardial infarction | X | X |
| COVID | 81-90 | w | 2 | Pneumonia |  | X |
| COVID | 91-100 | w | 3 | Pneumonia |  | X |
| COVID | 61-70 | m | 6 | ARDS | X |  |
| COVID | 51-60 | w | 4 | Pneumonia | X |  |
| COVID | 71-80 | w | 8 | Pneumonia | X |  |
| COVID | 71-80 | m | 6 | Pneumonia | X |  |
| COVID | 71-80 | w | 7 | Renal failure | X |  |
| COVID | 51-60 | m | 2 | Pneumonia | X |  |
| COVID | 71-80 | m | 2 | Pneumonia | X |  |
| COVID | 61-70 | m | 6 | Sepsis, Pneumonia | X |  |
| COVID | 61-70 | m | 4 | Pneumonia | X |  |
| COVID | 71-80 | m | 2 | Myocardial infarction | X |  |
| COVID | 81-90 | w | 5 | Subdural hemorrhage | X |  |
| COVID | 81-90 | m | 4 | Pneumonia | X |  |
| COVID | 81-90 | m | 2 | Sepsis, Pneumonia | X |  |
| COVID | 61-70 | m | 2 | Pneumonia | X |  |
| Control | 81-90 | w | 3 | Acute cardiac decompensation with intestinal infection | X |  |
| Control | 61-70 | w | 2 | Pneumonia in combination with gastrointestinal bleeding | X |  |
| Control | 81-90 | w | 2 | Chronic pneumonia and subarachnoid hemorrhage due to cerebral artery aneurysm. | X |  |
| Control | 51-60 | m | 3 | Congestive pneumonia with multiply metastasized paraganglion | X |  |
| Control | 71-80 | w | 3 | Bronchopneumonia |  | X |
| Control | 71-80 | m | 6 | Tumor-associated multiorgan failure | X |  |
| Control | 71-80 | m | 12 | Myocardial infarction |  | X |
| Control | 61-70 | m | 6 | Hemorrhagic shock due to esophageal variceal bleeding |  | X |
| Control | 31-40 | m | 6 | Acute exacerbated COPD |  | X |
| Control | 11-20 | w | 3 | Intoxication |  | X |
| Control | 61-70 | w | 4 | Pneumonia |  | X |
| Control | 41-50 | m | 5 | Transection of spinal cord |  | X |
| Control | 71-80 | m | 0 | Suffocation by hanging |  | X |
| Control | 71-80 | w | 0 | Traumatic brain injury and suffocation |  | X |

PMI = *post-mortem* interval

**Sup Table 2. Patients’ demographics for analyses of clinical data.**

|  | Mild | Moderate | Severe | Critical | Lethal |
| --- | --- | --- | --- | --- | --- |
| N  (% female) | 118 (47) | 48 (69) | 55 (42) | 47 (53) | 55 (64) |
| Age, mean  (SD) | 46.84 (18.8) | 53.69 (17) | 67.67 (13.54) | 60.51 (15.85) | 70.73 (12.52) |
| Respiratory rate  (per minute) | 13.74 (1.63) | 14.18 (1.67) | 14.76 (2.27) | 16.18 (2.87) | 14.85 (3.25) |
| CRP  (mg L^–1^) | 16.59 (25.51) | 39.53 (37.67) | 88.05 (77.17) | 136.13 (105.16) | 128.06 (96.01) |
| Lactate  (mmol L^–1^) | 1.28 (0.6) | 1.33 (0.6) | 1.55 (0.7) | 1.51 (0.45) | 2.35 (1.45) |
| Leukocytes  ($\boldsymbol{\times}$ 10^6^ mL^–1^) | 6.9 (2.63) | 6.35 (2.59) | 7 (3.34) | 10.15 (5.77) | 12.47 (9.38) |
| Peripheral O_2_ saturation (%) | 97.28 (2.32) | 95.67 (2.84) | 93.63 (3.66) | 93.18 (6.65) | 93.31 (6.47) |
| Venous CO_2_ saturation (%) | 45.14 (7.18) | 43.16 (6.49) | 42.9 (5.61) | 43.63 (7.01) | 43.96 (8.5) |
| pH | 7.4 (0.05) | 7.39 (0.05) | 7.4 (0.06) | 7.37 (0.07) | 7.37 (0.09) |
| Venous O_2_ saturation (%) | 37.77 (22.58) | 38.73 (27.12) | 36.4 (17.56) | 47.84 (31.62) | 40.65 (37.38) |
| Heart rate  (per minute) | 84.51 (21.13) | 88.09 (12.02) | 87.2 (16.21) | 84.16 (19.44) | 90.85 (21.63) |
| Temperature  (°C) | 36.64 (0.77) | 37.08 (0.82) | 37.04 (0.93) | 36.74 (0.88) | 36.92 (1.23) |

**Sup Table 3. Odds ratio analysis for prediction of lethality in critical COVID-19.**

|  | Odds ratio | *P*-value | CI_95%_ low | CI_95%_ high |
| --- | --- | --- | --- | --- |
| Peripheral O_2_ saturation  (%) | 1.06 | 0.28 | 0.95 | 1.18 |
| Respiratory rate (per minute) | 0.9 | 0.03 | 0.82 | 0.99 |
| CRP  (mg L^–1^) | 1.06 | 0.24 | 0.96 | 1.16 |
| Leukocytes  ($\boldsymbol{\times}$ 10^6^ mL^–1^) | 1.06 | 0.15 | 0.98 | 1.14 |
| Lactate  (mmol L^–1^) | 1.14 | 0.09 | 0.98 | 1.32 |
| Venous CO_2_ saturation (%) | 0.96 | 0.53 | 0.85 | 1.09 |
| pH | 1.05 | 0.4 | 0.94 | 1.18 |
| Venous O_2_ saturation (%) | 0.88 | 0.07 | 0.78 | 1.01 |
| Interleukin-6  (ng mL^–1^) | 0.95 | 0.11 | 0.89 | 1.01 |

**Sup Table 4. Summary statistics of correlation analyses between respiratory rate and its physiological modulators.**

|  | r | *P* value |
| --- | --- | --- |
| Peripheral O_2_ saturation  (%) | –0.06 | 0.34 |
| Heart rate  (per minute) | 0.07 | 0.27 |
| Diastolic blood pressure  (mmHg) | –0.05 | 0.44 |
| Systolic blood pressure  (mmHg) | –0.12 | 0.07 |
| Temperature  (°C) | –0.03 | 0.66 |
| CRP  (mg L^–1^) | 0.11 | 0.09 |
| Leukocytes  $\boldsymbol{(\times}$ 10^6^ mL^–1^) | 0.03 | 0.6 |
| Lactate  (mmol L^–1^) | 0.1 | 0.16 |
| Venous CO_2_ saturation (%) | –0.02 | 0.78 |
| pH | –0.09 | 0.22 |
| Venous O_2_ saturation (%) | 0.23 | < 0.01 |
| Interleukin-6  (ng mL^–1^) | 0.02 | 0.74 |
